# Phytosterols: Nutritional Health Players in the Management of Obesity and Its Related Disorders

**DOI:** 10.3390/antiox9121266

**Published:** 2020-12-12

**Authors:** Teresa Vezza, Francisco Canet, Aranzazu M. de Marañón, Celia Bañuls, Milagros Rocha, Víctor Manuel Víctor

**Affiliations:** 1 Service of Endocrinology and Nutrition, University Hospital Doctor Peset, Foundation for the Promotion of Health and Biomedical Research in the Valencian Region (FISABIO), 46017 Valencia, Spain; vezza_ter@gva.es (T.V.); francisco.canet.1994@gmail.com (F.C.); amardema@alumni.uv.es (A.M.d.M.); 2 CIBERehd, Department of Pharmacology, University of Valencia, 46010 Valencia, Spain; 3 Department of Physiology, University of Valencia, 46010 Valencia, Spain

**Keywords:** bioactive compounds, phytosterols, antioxidant properties, anti-inflammatory properties, microbiota, obesity, metabolic disorders

## Abstract

Obesity and its related disorders, such as diabetes and cardiovascular risk, represent an emerging global health issue. Even though genetic factors seem to be the primary actors in the development and progression of these diseases, dietary choices also appear to be of crucial importance. A healthy diet combined with physical activity have been shown to ameliorate glycaemic levels and insulin sensitivity, reduce body weight and the risk of chronic diseases, and contribute to an overall improvement in quality of life. Among nutrients, phytosterols have become the focus of growing attention as novel functional foods in the management of metabolic disorders. Phytosterols are natural plant compounds belonging to the triterpene family and are structurally similar to cholesterol. They are known for their cholesterol-lowering effects, anti-inflammatory and antioxidant properties, and the benefits they offer to the immune system. The present review aims to provide an overview of these bioactive compounds and their therapeutic potential in the fields of obesity and metabolic disorders, with special attention given to oxidative stress, inflammatory status, and gut dysbiosis, all common features of the aforementioned diseases.

## 1. Introduction

Over the last decade, obesity has become a global epidemic affecting both children and adult populations, with prevalence increasing more than four-fold since 1975. In 2016, more than 1.9 billion adults of 18 years and older were overweight, of which more than 650 million were obese [1]. This disorder has been associated with lower life expectancy and increased morbidity. In this regard, obesity has been shown to be the pivotal player in the physiopathology of both type 2 diabetes (T2D) and its cardiovascular complications [2]. Particularly, the excessive energy intake and low expenditure that characterise obesity lead to an energy imbalance and an abnormal accumulation of lipids in metabolic tissues, mainly liver and adipose tissue [3,4,5]. This results in the development of a low-grade systemic inflammatory state, which is related to the secretion of pro-inflammatory mediators such as interleukin (IL)-6 and tumour necrosis factor (TNF)-α. This promotes the recruitment of macrophages to adipose tissues and contributes to metabolic dysfunction, such as insulin resistance, impaired glucose tolerance, hyperglycaemia, and dyslipidaemia. In parallel, chronic hyperglycaemia induces oxidative stress via mitochondrial dysfunction and increased reactive oxygen species (ROS) generation or activated apoptosis in β-cells [6]. This elicits an increased pro-inflammatory response and leads to the onset of hypertension, endothelial dysfunction, high cardiovascular risk, and other obesity-related diseases [7]. Additionally, growing data have shown that the gut microbiota bears a significant functional role in the onset of metabolic disorders [8]. These diseases have been associated with an altered intestinal microbiota composition, also known as dysbiosis, along with increased gut permeability, both of which favour bacterial endotoxin translocation into the systemic circulation, thus contributing to the low-grade systemic inflammation reported in obese and diabetic subjects [9,10].

Lifestyle interventions consisting of restricted energy intake and more physical activity have been shown to improve metabolic and cardiovascular functions in obesity-affected subjects [11]. Nonetheless, most individuals do not adhere to such interventions, thus emphasising the need for other treatment modalities. To date, different pharmacological strategies have been used in the management of these diseases, including metformin, Glucagon-like peptide-1 receptor (GLP-1) agonists, amphetamine derivatives, and melanocortin-4 receptor agonists. Nevertheless, the use of these drugs is frequently problematic due to their side effects, such as dryness in the mouth, headaches, insomnia, and nausea, which are generally mild-to-moderate in intensity and wane if the therapy is prolonged [12,13]. Thus, due to the poor tolerability and lack of efficacy of the treatments used, along with the high costs of some of these drugs, there is currently considerable interest in investigating and developing options for natural products. These include dietary supplements (nutrients), herbal products, and processed foods such as beverages, soups, and cereals, that other than nutrition are also used as medicine [14]. 

Interestingly, growing evidence suggests plant-based diets as an alternative and/or complementary therapy for obese and diabetic patients, mainly due to their efficacy and safety and the greater sense of control they afford [15,16]. 

However, as most evidence has an empirical basis, it is important to assess the impact of natural products to confirm their use as prevention mechanisms or a convenient therapy for metabolic diseases. The literature extensively reveals that these phytochemicals have many active compounds responsible for their physiological functions; of note, they can act simultaneously against the different elements of the oxidative stress, inflammatory response, and gut dysbiosis, thus increasing their efficacy [17,18]. Among the compounds in question, sterols are thought to play a prominent role. These natural plant compounds, belonging to the triterpene family and structurally similar to cholesterol, are known to have cholesterol-lowering effects and antioxidant and anti-inflammatory properties, as well as affording benefits to the immune system. The present review aims to provide an overview of these phytochemical compounds from a nutritional perspective, and to highlight their therapeutic potential against obesity, diabetes, and related disorders, with special attention given to adipose tissue metabolism and inflammatory status, oxidative stress, mitochondrial dysfunction, and gut dysbiosis, all common features of said diseases. In conclusion, we hope to provide a valuable reference for studies related to obesity and diabetes in which phytosterol-based diets are assessed.

## 2. Phytosterols in Human Nutrition

Phytosterols are important micronutrients structurally similar and functionally analogous to cholesterols. Up to now, more than 250 phytosterols have been identified, with β-sitosterol, stigmasterol, and campesterol being the most commonly found in the diet. As summarised by Trautwein et al., they are essential structural components of the cellular membrane and serve crucial functions, such as modulating membrane permeability and fluidity [19]. Interestingly, they have attracted a great deal of attention due to their human health benefits, including antioxidant and cholesterol-lowering effects [17,18], in addition to their putative contribution to reducing the risk of cardiovascular diseases. Unlike cholesterol, humans cannot endogenously synthesise phytosterols and can obtain them only through diet. They are found in fruits, vegetables, beans, nuts, legumes, whole grains, tubers, wheat germ, vegetable oils, and sunflower seeds [20,21,22,23,24,25,26,27,28] (Table 1). 

**Figure 1:**
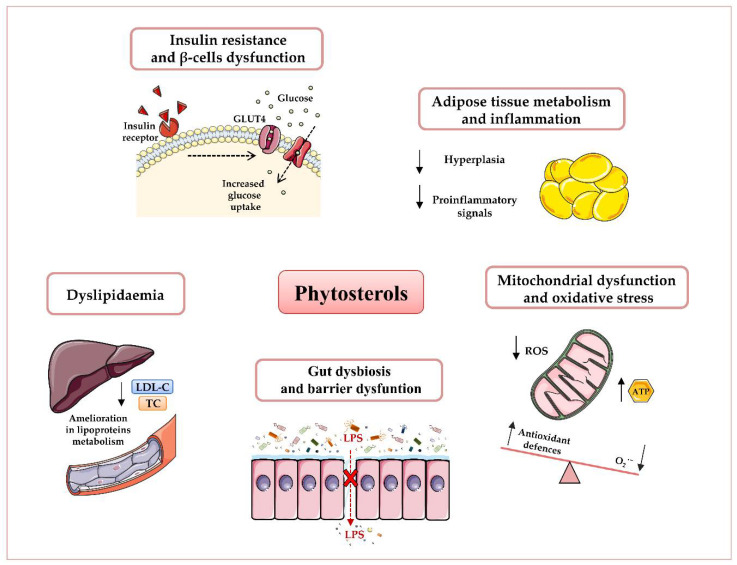
Potential beneficial effects of phytosterols in obesity and type 2 diabetes T2D prevention and therapy. Plant sterols have been shown to reduce dyslipidaemia, insulin resistance, and β-cells’ dysfunction, attenuate adipose inflammatory signalling, enhance mitochondrial ATP content, and decrease oxidative stress, as well as ameliorate gut microbiota dysbiosis and barrier dysfunction. Abbreviations: Adenosine triphosphate (ATP), Glucose transporter type 4 (GLUT4), Low-density lipoprotein-Cholesterol (LDL-C), Lipopolysaccharide (LPS), Reactive oxygen species (ROS), Total Cholesterol (TC).

A recent European study on phytosterols-enriched food customers’ purchase behaviour revealed that the daily intake of these compounds ranges from 200 to 400 mg in the general population, and 500–1000 mg in vegetarians [29], for whom variation depends upon the dietary habits and geographical areas. However, the European Food Safety Authority has pronounced that the beneficial effects of these compounds are obtained at high doses (around 2 g/day) that cannot be achieved through habitual diets. Hence, it is important to review and examine the dietary sources of phytosterols and to identify potential foods that are the key to improving phytosterol intake. Interestingly, growing evidence suggests phytosterols as nutritional modulators of immune response, mitochondrial dysfunction, oxidative stress, dyslipidaemia, and gut dysbiosis [30,31,32,33] (Figure 1). 

These benefits are mainly due to their antioxidant properties, by which they act directly, as free radical scavengers, or indirectly, by interfering with specific proteins in the redox signalling pathways implicated in various physiological actions [34]. Since phytosterols’ current relevance makes them the focus of much further research, the following sections provide readers an overview on their health benefits regarding common features of obesity and T2D. 

### 2.1. Effects of Phytosterols on Adipose Tissue Metabolism

Obesity is a complex and multifactorial disease that typically contributes to a cluster of disorders, including insulin resistance, hyperglycaemia, dyslipidaemia, and hypertension, which are closely associated with the rising incidence of T2D, cardiovascular disease, and stroke [7,35]. 

Adipose tissue, which is mainly composed of pre-adipocytes, adipocytes, fibroblasts, macrophages, leukocytes, and endothelial cells, has been widely identified as the key player in the regulation of systemic metabolism. Responding dynamically to hormonal and nutritional inputs, adipose tissue works as a source of energy-rich fatty acids during periods of negative energy balance in order to reduce lipid accumulation and release fatty acids to target tissues when energy is required [36]. There is much evidence that overnutrition can cause hypertrophic expansion of adipocytes, which, in turn, initiates a cascade of signalling and metabolic events leading to increased angiogenesis, tissue remodelling, extracellular matrix overproduction, and resident and non-resident immune cell recruitment and activation [37,38]. In this sense, some of these activated immune cells, namely macrophages (Mφ), polarise from a “classical” phenotype into a pro-inflammatory one, which activates a self-reinforcing cycle of high amounts of pro-inflammatory signals (such as TNF-α, IL-8, IL-6, and IFN-γ), in the adipose tissue and further progression of the inflammatory state. Persistent and exacerbated inflammation adipose tissue leads to hypoxia that stimulates hypoxia-inducible factor (HIF) 1α, thus increasing the adipose tissue fibrosis [39]. Concurrently, hypertrophic adipocytes stimulate lipolysis and raise levels of circulating FFA in the bloodstream. Thus, FFA can directly enter the liver via the portal circulation, thus inducing hepatic gluconeogenesis and insulin resistance [40]. Studies in both animals and humans showed that high levels of circulating FFA can lead to peripheral insulin resistance [40,41] as well as stimulate cytokine production of macrophages [42] and act as ligands for the toll-like receptor 4 (TLR4) complex [43]. Thus, they can modulate inflammation of adipose, thus contributing to obesity-associated metabolic comorbidities. Although circulating FFA levels do not predict the development of metabolic syndrome and do not increase in proportion to fat mass [44], several evidences suggest the correlation among the release of FFA from adipose tissue and the risk of obesity-associated complications [44,45].

In addition, adipocytes are extremely resistant to apoptosis inducers; therefore, adipose tissue mass expansion due to hyperplasia becomes chronic, making it difficult for an obese subject to sustain weight loss, and thus worsening the prognosis of its management [46,47,48]. Consequently, targeting hyperplasia is a crucial step in preventing the progression of childhood and adult obesity. Although relatively little research exists on the effects of phytosterols on adipose cells, research into these bioactive compounds has grown steadily over the last decade. Thornton et al. showed that animals treated with plant sterols experienced a dose-dependent reduction in body mass accumulation [40], with no side effects on gross morphology, muscle mass, organ mass, or femur length. In this regard, Rideout et al. suggested that this reduction in body weight gain could be associated with decreased fat absorption following consumption of phytosterols [49]. Particularly, recent data also demonstrate that oryzanol, an ester mixture of ferulic acid and phytosterols present in rice bran oil, reduces the body weight of Wistar obese rats in a dose-dependent manner [50].

In addition, many in vitro studies, performed mainly in 3T3-L1 cells (a mice-derived preadipocyte cell line), reveal beneficial effects of phytosterols on adipose tissue metabolism. Specifically, Lee et al. [51] reported that saringosterol, a steroid isolated from an edible brown alga (known as *Sargassum muticum*) distributed on the seashores of eastern and southern Korea, exerts anti-obesity effects by triggering the expression of several adipogenic genes, such as resistin, adiponectin, fatty acid synthase, and adipocyte fatty-acid-binding protein, in 3T3-L1 cells [51]. 

Moreover, the same study showed that saringosterol-mediated suppression of adipocyte differentiation is ascribed to its impact on two important transcriptional factors, namely CCAAT/enhancer binding protein α (C/EBPα) and peroxisome proliferator activator receptor γ (PPARγ), which orchestrate the expression of adipogenic and lipogenic genes. Indeed, while C/EBPα stimulates terminal adipocyte differentiation, PPARγ induces lipoprotein lipase expression and promotes fatty acid uptake [52]. As a result, the acetyl-coenzyme A carboxylase (ACC) transforms acetyl-CoA into malonyl-CoA, an inhibitor for fatty acid oxidation and a building block of fatty acid biosynthesis, and the transcriptional factor sterol regulatory element-binding protein 1c (SREBP-1c) that enhances adipogenesis and lipogenesis [51]. 

Similarly, β-sitosterol, one of the most abundant dietary phytosterols, has been extensively studied and found to possess beneficial properties, due to the reduction in adipose tissue mass and inhibition of preadipocytes proliferation [53]. Of note, β-sitosterol supplementation produced an important decrease in the growth of 3T3-L1 cells. This reduction was 12% to 19% at 2 mM, 24% at 8 mM, and 65% at 16 mM [53]. In addition, fucosterol, a sterol isolated from *Ecklonia stolonifera*, a brown alga, has been shown to inhibit 3T3-L1 adipogenesis by reducing the accumulation of lipid droplets, downregulating the PI3K/Akt and ERK pathways and, in turn, modulating the forkhead box protein O (FoxO) transcription factor [54], which have been reported to be associated with cell proliferation, differentiation, metabolism, and apoptosis. Particularly, fucosterol was also shown to regulate adipogenic differentiation through activation of the Wnt/β-catenin signalling pathway as well as its major components β-catenin, cyclin D1 (CCND1), and dishevelled 2 (DVL2) [55].

Growing clinical trials further support the anti-obesity effects of these phytosterols in patients with obesity. Takeshita et al. reported a decrease in visceral, subcutaneous, and abdominal adipose tissue after phytosterol consumption in Japanese men [56]. Moreover, a cross-sectional Chinese study of 503 women and 409 men showed that high dietary phytosterol intake is related to a low body mass index (BMI) [57]. In 2019, data from randomised controlled trials demonstrated that phytosterol supplementation (1 and 2 g during ≥16 weeks) reduced BMI and body weight significantly when baseline BMI was higher than 25 [58]. 

Unfortunately, the exact mechanisms that may explain the positive effects of phytosterols on adipose tissue expansion and metabolism are not clearly understood and further studies are needed, also to investigate the adequate dose to evoke a substantial anti-obesity effect in clinical settings, mainly due to the conflicting results between the clinical trials and in vitro studies. Indeed, because phytosterols are poorly absorbed in the intestine (0.4–3.5%), a higher dose cannot lead to an elevated absorption. Nevertheless, it was shown that a moderate and regular dose of sterols given over a long period of time can result in higher levels and better effects of these compounds [59]. 

### 2.2. Effects of Phytosterols on Adipose Tissue Inflammation

Besides storing excess energy in the form of triglycerides, adipose tissue exerts a dynamic endocrine function. It releases many active mediators, named adipocytokines—including TNF-α, IL-6, resistin, and leptin—that participate in the regulation of several biological functions, such as energy homeostasis, systemic metabolism, and inflammation [60]. Increased visceral adiposity and adipocyte dysfunction can promote the dysregulation of these mediators, thus leading to local and generalised inflammation [61]. In particular, TNF-α activates proinflammatory signalling cascades, such as AP-1 (activator protein-1) and mitogen-activated protein kinase (MAPKs), which downregulate the master regulators of adipogenesis, known as peroxisome proliferator-activated receptors (PPAR), and induce the transcription of inflammatory genes [61]. Concurrently, pro-inflammatory macrophages are mostly recruited from circulating monocytes into fat tissue, whereas resident classically activated macrophages proliferate and differentiate in crown-like structures, switch into a pro-inflammatory profile, and then move throughout adipose tissue [62]. These processes are facilitated by several mobilisation profiles of other crucial players in inflammation, namely T lymphocytes (T helper (Th)1, Th2, and Treg). Particularly, the number of Treg (Foxp3^+^) and Th2 (CD4^+^ GATA3^+^) anti-inflammatory T cells decreases, whereas the number of cytotoxic T cells (CD8^+^) increases [63]. As a result, this persistent inflammatory condition leads to an impairment of insulin signalling, as well as exacerbating beta (β)-cell dysfunction and obesity-triggered insulin resistance [5,53], along with abnormal tissue remodelling and fibrosis. Specifically, the inflammatory stimuli activate c-Jun *N*-terminal kinase (JNK), a protein kinase expressed in myeloid and insulin-targeted cells [64], that inhibits the insulin signalling pathway through inhibitory serine–threonine phosphorylation of the insulin receptor substrate-1 (IRS-1), thereby decreasing PI3K/PKB signalling [65] and leading to insulin resistance. 

Growing evidence has shown the ability of phytosterols to interfere with inflammatory signalling through different pathways. For example, campesterol, β-sitosterol, ergosterol, and stigmasterol have been revealed to inhibit the production of inflammatory enzymes and proinflammatory cytokines in different cell lines. Particularly, β-sitosterol isolated from *Moringa oleifera* reduced the secretion of TNF-α, IL-8, IL-1β, and IL-6 in LPS-stimulated HaCaT human keratinocytes and J774A.1 mouse macrophages [66]. This sterol was also able to decrease the expression of the inflammatory NLRP3 (nucleotide oligomerisation domain (NOD), leucine-rich repeat (LRR), and pyrin domain (PYD)), thus inhibiting the activation of the protease caspase-1 [66], which normally cleaves the pro-form of different cytokines (IL-18, IL-1β, and IL-33) and generates their mature secreted forms that improve the inflammatory response [67]. Besides, saringosterol, isolated from *Sargassum muticum*, was shown to inhibit the adipocyte differentiation by dose-dependently reducing expression of adipogenic marker genes such as resistin, adiponectin, adipocyte fatty acid-binding protein, and fatty acid synthase in 3T3-L1 cells [51]. 

It is important to note that one of the primary mediators of stress and inflammatory responses linked to obesity is the nuclear factor kappa-light-chain-enhancer of activated B cells (NF-κB) [68]. This is a protein complex that controls the transcription of DNA, cytokine production, and cell survival, and which can play a key role in regulating the immune response. It is well established that, under normal conditions, the NF-κB complex is present in the cytoplasm in an inactive state. However, during obesity, multiple means such as FFAs, microbiota-derived lipopolysaccharide (LPS), inflammatory cytokines, advanced glycation end products (AGEs), endoplasmic reticulum (ER) stress, and oxidative stress, can recruit inflammatory signalling cascades that can interfere with different NF-kB molecules. Specifically, they can activate NF-κB kinase subunit 2 (IKK2) and degrade the inhibitory IκB-α protein by directly binding to toll-like receptor 4 (TLR4), a member of the toll-like receptor (TLR) family, which acts as a crucial player in the activation of innate immunity and pathogen recognition. As a result, the activated NF-κB complex is now free to translocate to the nucleus, where it promotes the transcription of specific immune response genes [68,69]. Additionally, it is widely accepted that overnutrition is closely related to increased intestinal inflammation and circulating LPS concentration (known as metabolic endotoxemia), which, in turn, promotes an increment in TLR4 expression and NF-κB activation [68]. In accordance with the initiation and progression of an inflammatory state, FFAs activate the serine kinase IKK, which drives the functional inhibition of two molecules that regulate the metabolism of glucose and lipids—the peroxisome proliferator activated receptor (PPAR)γ and the insulin receptor substrate-1 (IRS-1)—thus leading to a reduction in insulin sensitivity [68]. 

Of note, in addition to the above-mentioned NF-κB translocation-dependent pathway, which involves IκB degradation and IKK-dependent phosphorylation, NF-κB activation is also mediated by a second pathway, which includes signalling molecules, namely phosphorylations of the mitogen-associated protein kinases (MAPKs), such as the stress-activated protein kinases (p38/SAPKs), the Jun amino terminal kinases (JNKs), and the extracellular signal-regulated protein kinases (ERKs) [70]. While the ERKs induce cell differentiation and growth by responding primarily to mitogens and growth factors, the JNKs are involved in metabolism, cytokine production, inflammation, and apoptosis, and respond to stress such as oxidative stress and ionising radiation. Similarly, the activation of p38 MAPKs by cytokines and cellular stress contributes to cell cycle regulation, cell differentiation, inflammation, and apoptosis [71]. Interestingly, many researchers have demonstrated that obese subjects show an activated phosphorylation of JNK that negatively regulates the insulin signalling pathway. Indeed, upon activation, JNK is translocated from the cytoplasm into the nucleus, thus promoting the expression of several pro-inflammatory genes and protein synthesis (e.g., IL-1β, TNFα, IL-8, and IL-6), impairing glucose tolerance by obesity-induced insulin resistance [72]. Similarly, inappropriately increased activation of p38 MAPK has been suggested to contribute to insulin resistance by downregulating the expression of the insulin-responsive glucose transporter (GLUT4) and, in turn, reducing insulin-stimulated glucose uptake [73].

Subsequent research demonstrated that inhibition of these stress-activated mechanisms enhances the obesity-induced inflammatory status. In this regard, several plant-derived compounds were found to suppress NF-κB-dependent gene expression by modulating both pathways. For instance, β-sitosterol is able to attenuate experimental high-fat diet-induced intestinal inflammation by inhibiting LPS binding to TLR4 in the NF-κB pathway [74], data confirmed by studies in LPS-stimulated intestinal macrophages [74]. Further studies performed in the same cell line showed that fucosterol inhibit the LPS-induced CCAAT/enhancer binding protein b (C/EBPb) and DNA-binding activity of NF-κB, as well as the phosphorylation of p38, JNK, and ERK MAPK [75]. These results are similar to those obtained for other phytosterols, such as stigmasterol [76] and β-sitosterol [77], both of which are able to inhibit the LPS-induced NF-κB signalling cascade in macrophages. 

In terms of clinical trials, Lambert et al. showed that 4 weeks of phytosterol-supplemented milk intake (1.6 g/250 mL of milk) decreased the inflammatory state in obese patients [30] by suppressing the expression of the monocyte chemotactic protein-1 (MCP-1), a critical chemokine that modulates the infiltration and migration of macrophages and monocytes to the inflamed tissue. Phytosterol supplementation was also able to suppress the expression of interleukin-10-R and C–C motif chemokine 2, two essential cytokines with anti- and pro-inflammatory properties respectively, in these obese patients [30]. This finding has been confirmed in further experiments showing that β-sitosterol prevents obesity-associated low-grade inflammation by decreasing circulating IL-6 and TNF-α [78]. However, the underlying mechanisms by which phytosterols ameliorate the expression and release of these inflammatory markers are still poorly understood and further research is warranted to clarify them. 

### 2.3. Effects of Phytosterols on Oxidative Stress

The increasing data have revealed a close link between obesity, T2D, oxidative stress, and mitochondrial dysfunction [79,80]. First of all, it is important to specify that ROS are oxygen-derived molecules essentially produced by NO**^•^**, NADPH oxidase, and mitochondria in response to bacterial invasion or cytokines [81]. Included among ROS are hydrogen peroxide (H_2_O_2_), superoxide anion (O_2_^−^), and hydroxyl radicals (**^•^**OH) [82], all of which can act as secondary messengers participating in many biological processes, such as differentiation, proliferation, apoptosis, and the immune response [82]. In adipose tissue, ROS contribute to adipocyte differentiation through H_2_O_2_-induced cyclic adenosine monophosphate (cAMP) response element-binding protein beta (C/EBP-beta) DNA-binding activity, and to preadipocyte proliferation metabolic homeostasis [83,84]. It is well known that, under physiological states, oxidative cellular homeostasis ensures a balanced equilibrium between ROS generation and antioxidant defence systems. Particularly, these systems include enzymatic and nonenzymatic antioxidants, including glutathione peroxidase (GPx), Kelch-like ECH-associated protein 1 (Keap1)-NRF2-ARE, or superoxide dismutase (SOD) and catalase, which neutralise the harmful effects of ROS [85]. However, overproduction of ROS can lead to an imbalance between the generation and inactivation of these molecules, thus leading to mitochondrial and adipocyte dysfunction. Thus, these uncontrolled events result in lipid accumulation and the activation of cytosolic signalling pathways that facilitate adipose tissue inflammation, impaired adipogenesis, and insulin sensitivity [84,86,87]. In this sense, a body of data is testimony to the disruptive and chronic oxidative stress damage generated in obesity and T2D [88,89]. Overnutrition can stimulate the release of inflammatory markers in adipose tissue, thus resulting in systemic inflammation and insulin resistance, hyperinsulinemia, and related complications. Of note, the altered expression and production of pro-inflammatory cytokines can lead to the onset of oxidative stress [90], mainly via catalytic activity of the nicotinamide adenine dinucleotide phosphate (NADPH) oxidase (NOX) enzyme [88,91]. NADPH oxidase-derived ROS facilitate the recruitment of monocytes and macrophages from the bloodstream to the inflamed tissue, and consequently the development of insulin resistance and systemic inflammation. In particular, ROS liberate TXNIP (thioredoxin-interacting protein), a crucial protein associated with insulin resistance, which stimulates the assembly of a cytosolic multiprotein complex, namely NLRP3 (nucleotide oligomerisation domain (NOD), leucine-rich repeat (LRR), and pyrin domain (PYD)) inflammasome, that promotes inflammation. In this sense, NLRP3 activates procaspase-1, which, in turn, cleaves pro-IL-18 and pro-IL-1β into their mature bioactive forms [92]. These pro-inflammatory molecules released into the systemic circulation lead to the induction and propagation of the chronic inflammatory state that characterises obesity and T2D [93].

In summary, oxidative stress produced under obese and diabetic conditions can generate mitochondrial dysfunction and, by influencing each other, they lead to further tissue damage. Although several drugs that target mitochondria and oxidative stress are currently under investigation for the treatment of metabolic disorders, their prolonged use may cause harmful side effects. Therefore, the potential of pharma-nutrition for the management of these pathologies has attracted a lot of attention. Of note, although only a few data are currently available, many investigations have suggested that mitochondria and oxidative stress constitute potential direct or indirect targets of phytosterols. In this context, sitostanol and sitosterol show beneficial effects on mitochondrial respiration in human hepatocytes, brown adipocytes, and myotubes [94]. Moreover, sitosterol has been shown to enhance mitochondrial ATP content and membrane potential in H9c2 cardiomyocytes [95]. Specifically, this sterol induces mitochondrial uncoupling, thus triggering a retrograde upregulation of cellular antioxidant components to protect against hypoxia/reoxygenation (Hypox/Reoxy)-induced apoptosis in these cells. Yoshida et al. demonstrated that sitosterol can also reduce oxidative damage in streptozotocin-induced experimental hyperglycaemia [96]; indeed, treatment with this sterol (10, 15, and 20 mg/kg per day) reduced serum NO levels in diabetic rats, thus endorsing its free radical-scavenging capacity [96]. Previous studies performed in RAW 264.7 mouse macrophage cultures also evidence the capacity of this sterol to revert the impairment of the glutathione/oxidised glutathione ratio induced by phorbol esters [97]. The authors in question showed that the effect of this compound on antioxidant enzymes was mediated by the oestrogen/phosphatidylinositol 3-kinase pathway [97]. Meanwhile, studies performed on INS-1 insulinoma cells and human islets have shown that stigmasterol treatment can prevent β-cell dysfunction induced by glucolipotoxicity, an effect that was associated with decreased insulin secretion and increased ROS production [98].

Taken together, these results suggest that plant sterols act chemically as antioxidants and physically as a stabiliser of membranes. Further in-depth experiments on plant sterols and their associated mitochondrial membrane effects may clarify how exactly these compounds decrease ROS production as well as increasing ATP content and, in turn, mitochondrial membrane potential. 

### 2.4. Effects of Phytosterols on Blood Glucose and Insulin Resistance

Insulin is an anabolic peptide hormone secreted by pancreatic β cells and acting through an insulin receptor (IR) located in the membrane of target cells (liver, fat, skeletal muscle, brain cells, etc.). It serves many physiological activities: fat glucose and boost muscle uptake, lipid synthesis and storage in liver and fat, muscle and liver synthesis of glycogen and protein, and restrains glycogenolysis, gluconeogenesis, and fatty acid oxidation. Thus, insulin increases glucose uptake by reducing circulating glucose and lipid levels as well as increasing its conversion into fat or glycogen. 

Physiological insulin signalling is mediated by the binding and activation of IR, which evokes conformational changes and auto-phosphorylation that subsequently lead to the recruitment and phosphorylation of intracellular proteins called insulin receptor substrates (IRS) and Sh2-containing collagen-related protein (Shc) [99]. IRS proteins activate the PI3K-Akt pathway, whereas Shc activates the Ras-MAPK pathway. Specifically, IRS proteins recruit and activate the phosphoinositol 3 kinase (PI3K), leading to the production of phosphatidylinositol 3,4,5-triphosphate (PIP3), a second messenger that, in turn, recruits and activates 3-phosphoinositide-dependent protein kinase-1 (PDK-1). Upon activation, PDK-1 phosphorylates Akt, which regulates glucose uptake through the translocation of the insulin-sensitive glucose transporter GLUT4 to the cell membrane of muscle and fat cells. Meanwhile, Akt also mediates and regulates the lipid synthesis, gluconeogenesis, glycogen synthesis, and other insulin metabolic effects [99].

Similarly, insulin signalling can stimulate the recruitment and phosphorylation of Shc, which, consequently, lead to the activation of rat sarcoma protein (Ras) via the Grb2-Sos pathway, which is independent of PI3K/Akt. c-Raf, a serine-threonine protein kinase that phosphorylates and activates mitogen-activated protein kinase (MEK), binds to this activated Ras. Activated MEK catalyses phosphorylation of mitogen-activated protein kinase (MAPK) (also known as extracellular signal-regulated kinase, Erk). The phosphorylated Erk enters the nucleus, where it activates transcription factors associated with cell proliferation, protein synthesis, and cell division [99]. 

Growing data suggest that fat content, inflammation, and oxidative stress together can contribute to different hallmarks of metabolic syndrome, such as β-cell functional deterioration and insulin resistance. Of note, the relatively insufficient release of insulin by pancreatic β cells leads to defective metabolic insulin signalling and impaired glucose uptake into adipose tissue and skeletal muscle, along with an uncontrolled suppression of hepatic gluconeogenesis and glucose release into the bloodstream [100,101]. Interestingly, accumulating evidence shows that insulin resistance is characterised by downregulation of the major insulin-responsive glucose transporter, GLUT4, whose main role is to provide insulin-stimulated glucose uptake by different tissues and organs (skeletal muscle, adipose tissue, and the heart) that specifically express this protein [102]. This transporter seems to be the target of many phytosterols: it can induce GLUT4 translocation in skeletal muscle or adipocytes cells by modulating either AMP-activated protein kinase (AMPK) or insulin-mediated PI3-K/Akt pathways [103,104]. 

In this regard, stigmasterol has been shown to reduce insulin resistance in high-fat-fed KK-Ay mice [105]. When these mice were administered 50 mg/kg/day of this compound, an improvement in glucose tolerance was observed after 4 weeks. This beneficial effect could have been due to the ability of this sterol to normalise the forms of islets and prevent a decrease in pancreas islet size, as well as increasing GLUT4 expression levels in skeletal muscle and white adipose tissue. Furthermore, rats administered β-sitosterol presented an improved oral glucose tolerance, reduced fasting glucose levels, and increased insulin secretion from isolated rat pancreatic islet cells [106,107]. Further research confirmed that these effects are mediated by the activation and regulation of PI3K/Akt and GLUT4 [108]. Commonly, the phytocompound exerted a significant level of lipolytic effect at lower concentrations but diminished gradually at higher concentrations. These findings accorded with the real time polymerase chain reaction (RT-PCR) results: GLUT4 gene expression was upregulated, whereas PI3KA and AKT genes were downregulated [108]. Moreover, histological observations point to the rejuvenation of insulin-producing β-cells in sitosterol-treated diabetic rats [96]. In this study, 30-day oral administration of sitosterol (20 mg/kg) to a high-fat diet and sucrose-induced T2D rats normalised the impaired plasma insulin and blood glucose levels [96].

In addition, different in vitro studies have confirmed the ability of phytosterols to enhance glucose homeostasis. Particularly, accumulating evidence shows that β-sitosterol and stigmasterol are capable of increasing adipocyte glucose uptake and GLUT4 translocation and expression in L6 cells [105], and immortalised rat skeletal (L6) myoblast cells with endogenous expression of GLUT4 and high fusion potential in the myotube stage. This beneficial effect of phytosterols on glucose metabolism seems to be mediated by AMPK [109]. 

Collectively, these findings show that phytosterols may target the leading cause of insulin resistance and thereby contribute to the overall amelioration of metabolic hallmarks in these pathologies. However, the evidence obtained in humans is limited and further in-depth studies are required to shed light on their intricate mechanisms of actions. 

### 2.5. Effects of Phytosterols on Obesity-Related Dyslipidaemia 

Dyslipidaemia is considered to be one of the critical components of obesity, T2D, and their related disorders. It consists of an increased low-density lipoprotein (LDL)/very-low-density lipoproteins (VLDL)/triglycerides (TG), and decreased level of high-density lipoprotein (HDL) [110,111]. The precise pathogenesis of diabetic dyslipidaemia is unknown; nevertheless, different mechanisms seem to be responsible for the development of this condition, including insulin-controlled apoprotein production in the liver, actions of cholesteryl ester transfer protein (CETP), regulation of lipoprotein lipase (LPL), and peripheral actions of insulin on adipose tissue and muscle [112]. Among these, the enzyme LPL is thought to be a prominent actor. Indeed, it is widely accepted that under physiological circumstances, insulin activates LPL, thus hydrolysing triglycerides in FFAs, which is then taken up by cells. However, in obese subjects, elevated plasma insulin levels and insulin resistance can generate LPL dysfunction. As a result, there is increased esterification of hepatic FFAs, formation and storage of triglycerides in adipose tissue, and a subsequent increase in the production of glucose [110,111,113]. Moreover, FFAs inhibit insulin-mediated glucose uptake by reducing insulin sensitivity in muscles. On the other hand, high blood glucose concentration increases insulin secretion, leading to even more intensified hyperinsulinemia. These pathogenic conditions influence each other and promote the initiation and progression of T2D and coronary heart disease [112]. In addition, growing evidence reveals that dyslipidaemia is associated with increased levels of certain inflammatory mediators, including TNF-α and IL-6, which play a critical role in producing insulin resistance [114]. Interestingly, popular lipid-lowering drugs, like statins, do not efficiently address parallel pathologies such as insulin sensitivity and inflammation. Therefore, future research needs to shift its attention from synthetic to natural products, which can produce a more balanced therapeutic effect across inflammation/insulin resistance and obesity/dyslipidaemia. 

In this regard, over 40 clinical trials have evaluated the effects of plant sterols on several metabolic syndromes [31,115,116]. The consumption of 2 g/day of phytosterols in orange juice, margarine, olive oil, tablets, mayonnaise, yoghurt, or low-fat milk has been found to significantly reduce some inflammatory markers and LDL-C by 10–11% of baseline values over 1–12 months in children, T2D patients, and adults with normal or high cholesterol [116]. A randomised, double-blind trial investigating the effects of daily consumption of phytosterol-enriched milk on the lipid profile of children with dyslipidaemia also showed significant reductions in LDL-C and TC levels versus the skimmed milk group of 10.2% and 5.9%, respectively [117]. Data reported by Miettinen et al. showed that hypercholesterolaemic patients treated with sitostanol-ester margarine (2.6 g for 6 months) underwent an important reduction in LDL-C concentration levels at 12 months, averaging 14% of baseline, with no change registered in HDL-C and triglyceride levels [118]. Moreover, plant sterols have revealed additive effects when combined with statin treatment, mostly recommended in diabetes patients, leading to a decrease in the absorption of exogenous cholesterol and bile cholesterol [91]. The underlying mechanisms of these effects are unclear, although the most widely documented candidate is the inhibition of intestinal cholesterol absorption [119]. In brief, during lipid digestion, free cholesterol is solubilised in micelles and taken up into the enterocyte. Once taken up, cholesterol is generally esterified by intestinal Acyl-CoA:cholesterol acyltransferase 2 (ACAT-2), a microsomal protein responsible for intracellular cholesterol ester synthesis. These so-formed cholesteryl esters are then packaged into chylomicrons and transported to the hepatic circulation system through the lymphatic system. Interestingly, phytosterols compete with free cholesterol for the binding with the micellar structure, thus undermining cholesterol solubilisation and increasing cholesterol excretion via the faeces [120]. Moreover, many studies have highlighted the low affinity of plant sterols for ACAT-2, which facilitates their circulation in the free form [121]. A body of data suggests that phytosterols interfere with a crucial intestinal cholesterol sensor known as liver X receptor (LXR), which controls the transcription of lipid metabolism; after activation, it is implicated in cholesterol absorption, efflux, transport, and excretion [121]. 

Current knowledge is sufficient to recommend the inclusion of these compounds in the daily diet of adults and children in order to improve dyslipidaemia in adults and children. However, their long-term safety has not yet been confirmed, and further studies are required to endorse the significance of previous findings. Moreover, phytosterols are present in our food in combination with other nutritional compounds; thus, other dietary factors may also modulate the effects of sterols in the body. 

### 2.6. Effects of Phytosterols on Gut Microbiota

Accumulating data over the past decade suggest that obesity and T2D are related to an altered gut microbiota (known as dysbiosis) [122,123,124,125]. The gut microbiota is a highly complex bacterial community that colonises the gastrointestinal tract. It consists of up to 100 trillion microbes of more than 1000 different species that perform different critical functions: energy regulation, metabolization of xenobiotics, production of vitamins such as vitamin K, folate, and biotin, modulation of the development of a mature immune system preventing colonisation by pathogens, and fermentation of dietary fibres in short-chain fatty acids (SCFAs) [126]. Metagenomic studies in humans and mice have shown that the two most abundant gut bacterial phyla, *Bacteroidetes* and *Firmicutes* [127,128], maintain a relative balance in lean subjects. However, obese humans and rodents are characterised by an increased ratio of *Firmicutes* to *Bacteroidetes*. In particular, obese individuals present a reduction of *Akkermansia muciniphila*, a mucin-degrading bacterium that has been inversely related to glucose intolerance and body fat mass in both humans and mice, as well as an impairment in *Lactobacillus* and *Clostridium* species, which are correlated with insulin resistance [129,130]. These alterations in gut microbiota composition induced and increased intestinal permeability to bacterial LPS, resulting in elevated levels of systemic LPS that exacerbate the low-grade inflammatory status of obese individuals [128,131,132]. 

Since carrying out a controlled dietary intervention study in humans is not without difficulties, the complicated interplay between host genetic background, age, diet, and host environment in the regulation of intestinal microbial ecosystems is not completely understood. Nevertheless, different studies have suggested that modification of the gut microbiota by behaviour, including contemporary dietary habits, might provide a novel treatment target in the obesity plague and its comorbidities [133,134]. 

Currently, there is much scientific evidence to show that phytosterols exert a protective role against these pathologies by modulating gut microbiota composition and functionality, as well as having a beneficial impact on intestinal inflammation and barrier integrity. Impressive results were obtained during the in vitro fermentation process in human faeces, where sterol supplementation was found to decrease the abundance of *Erysipelotrichaceae*, a bacterial family observed in morbidly obese individuals and directly linked with lipidemic imbalances [32,33]. These microbial changes were also confirmed in mice and hamster models of hypercholesterolaemia [33]. Moreover, data in rats have shown that supplementation with stigmasterol [135] or β-sitosterol [136] results in an amelioration of intestinal dysbiosis. As a result, increased cholesterol and coprostanol excretion occurs, together with a decrease in esterified cholesterol liver levels and plasma non-HDL cholesterol. 

The above-mentioned reports were based on in vitro and in vivo models. Future research is warranted to clarify if these modulation patterns can be replicated in human studies, to thus determine whether the relationship between sterols and the gut microbiota is a direct or indirect effect of a plant-based diet. 

## 3. Conclusions

Obesity and diabetes have become global epidemic issues affecting both children and adult populations, and their prevalence has resulted in an increase of morbidity and mortality in recent years. Accumulating research suggests that phytosterols are an alternative and/or complementary therapy for obese and diabetic patients, mainly because of their efficacy, safety, and patients’ sense of control over their treatment/healthcare. Reported data attribute the beneficial effects of phytosterols to their ability to act as nutritional modulators of immune response, oxidative stress, adipose tissue metabolism, hypercholesterolemia, and gut dysbiosis. 

Although the collective knowledge about the health benefits of phytosterols in obesity and T2D is increasing, it is based on empirical evidence and the underlying mechanisms of action remain unclear. For example: “What specific molecular triggers govern the beneficial effects of phytosterols?” “Are their benefits mainly due to their acting directly as free radical scavengers, or do they indirectly interfere with specific proteins in the redox signalling pathways?” “How do phytosterols modulate gut microbiota composition and improve the intestinal barrier function?” “What are the pathways that have a positive impact on clinical outcomes?” “Which specific molecular mechanism helps plant sterols to relieve inflammation in adipose tissue and affect fat distribution in the body?”.

Further studies are necessary to answer these questions in order to sufficiently evaluate the impact of these natural drugs and confirm their role as prevention mechanisms or effective therapy for metabolic diseases. 

## 4. Survey Methodology 

A literature review was conducted aimed at collecting any published data about phytosterols and their potential action as novel functional foods in the management of obesity and its associated complications. Databases such as Google Scholar and PubMed were used for searching literature relevant to the topic of the article. Phytochemicals, phytosterols, plant-based diet, pharma-nutrition, oxidative stress, gut dysbiosis, type 2 diabetes, and obesity were used as keywords. Screened articles were used as references for this review. 

### Study Selection Criteria 

The inclusion criteria were as follows: (1) articles that showed the efficacy of phytosterols in any dosage form (injection, extract, functional foods, etc.), (2) reports that included in vitro, in vivo, or human experiments, (3) original research articles, (4) review articles, (5) articles and reviews written in English, and (6) articles published between 2000 and 2020. The exclusion criteria were: (1) articles and reviews on diseases other than obesity, type 2 diabetes, and cardiovascular diseases, and (2) articles on phytochemicals other than phytosterols. 

## Figures and Tables

**Table 1:** Phytosterols’ food sources and their content, expressed as mg/100 g of dry product.

Source		Phytosterols Content (mg/100 g Dry Product)	Reference
	**Oils**		
Refined olive oil		235.9	[21]
Virgin olive oil		259.7	[21]
Argan oil		188.1	[22]
Sunflower oil		492.5	[21]
	**Vegetables**		
Artichoke		48.5	[21]
Green asparagus		10.6	[21]
Green beans		18.8	[21]
Broccoli		18.3	[21]
Cabbage		27.4	[21]
Carrot		18.6	[21]
Cauliflower		44.3	[21]
Celery		7.8	[21]
Chard		16.6	[21]
Cucumber		7	[21]
Eggplant		5.9	[21]
Endive		16.9	[21]
Escarole		18.1	[21]
Garlic		18.2	[21]
Leek		11.7	[21]
Lettuce		13.5	[21]
Marrow		2.4	[21]
Onion		7.2	[21]
Parsley		7.4	[21]
Potato		4.3	[21]
Green pepper		9.4	[21]
Red pepper		7.4	[21]
Spinach		16.3	[21]
Tomato		9.9	[21]
	**Cereals**		
Rice		29	[21]
White wheat		41.9	[21]
Wheat grain		315.7	[21]
Wheat bran		459	[21]
Wheat flour		140	[21]
Barley		130.8	[23]
Rice bran		450	[21]
Corn bran		300	[21]
Oat bran		150	[21]
	**Legumes**		
Chickpea		121.1	[21]
Lentil		117.3	[21]
White bean		108.1	[21]
Peanuts		406	[24]
	**Fruit**		
Apple		16	[21]
Apricot		15.2	[21]
Banana		20.1	[21]
Cherry		20.1	[21]
White grape		32.6	[21]
Kiwi		7.1	[21]
Melon		3.3	[21]
Olive		37.7	[21]
Orange		30.4	[21]
Peach		14.6	[21]
Pear		11	[21]
Plum		18.9	[21]
Strawberry		11.3	[21]
Watermelon		4.5	[21]
Avocado		25.5	[25]
Pineapple		4.9	[25]
Apple		5.31	[25]
Custard apple		62.3	[26]
Raspberry		25	[27]
	**Nuts**		
Almond		148.6	[21]
Hazelnut		128.1	[21]
Peanut		143.6	[21]
Pistachio		242.7	[21]
Sunflower seed		226.9	[21]
Walnut		131.3	[21]
Brazil nuts		95	[28]
Cashews		150	[28]
Macadamia nuts		187	[28]
Pecans		157	[28]
Pine nuts		236	[28]
